# Expanding our food supply: underutilized resources and resilient processing technologies

**DOI:** 10.1002/jsfa.13740

**Published:** 2024-07-11

**Authors:** Dietrich Knorr, Mary Ann Augustin

**Affiliations:** ^1^ Food Biotechnology and Food Process Engineering Technische Universität Berlin Berlin Germany; ^2^ CSIRO Agriculture and Food Werribee Victoria Australia; ^3^ School of Agriculture, Food and Wine University of Adelaide Urrbrae South Australia Australia

**Keywords:** food security, biodiversity, diet, sustainability, processing, consumer

## Abstract

Many underutilized food resources have been traditionally used by regional and poor communities. The history of their consumption makes them potential new food sources for incorporation into the wider food supply. The ability to tap the potential of undervalued and underutilized food sources will reduce the world's reliance on a limited number of food sources and improve food security and sustainability. The expansion of the food diversity of the food supply to include underutilized food resources will require overcoming challenges in the efficient and profitable production of the raw material, application of suitable postharvest handling procedures to maintain the quality of perishable produce, and the use of appropriate traditional and emerging food processing technologies for conversion of the raw material into safe, nutritious and consumer‐acceptable foods. Improvement of food processing technologies, particularly resource‐efficient resilient food processes, are required to ensure the safety, quality and functionality of the whole food or extracts, and to develop ingredient formulations containing new foods for manufacture of consumer food products. Factors that help facilitate the social acceptance of new underutilized foods include increasing consumer knowledge and understanding of the contribution of new underutilized food resources to diet diversity for good nutrition, confidence in the safety and value of new foods, and their low environmental impact and importance for future sustainable food. The introduction of new underutilized food resources will increasingly require collaboration along the whole food value chain, including support from government and industry. © 2024 The Author(s). *Journal of the Science of Food and Agriculture* published by John Wiley & Sons Ltd on behalf of Society of Chemical Industry.

## INTRODUCTION

Of the 6000 plant species that humans have been eating over time, the world now mostly eats just nine with rice, wheat and maize providing 50% of all calories.^1^ With the advent of technology, the world began producing foods more efficiently. The focus has been on high yielding uniform food products from crops and livestock. With the increased adoption of monocultures for agricultural food production during the green revolution, the focus was on the production of calories to feed the world without much consideration paid to biodiversity, many indigenous varieties of crops, soil health, greenhouse gas emissions, environmental degradation, and food security.^1^, ^2^, ^3^ There is a need to expand the food supply and stem the dependence of the global food system on a limited number of food sources. This requires a food systems approach which promotes sustainable production, processing and consumption which harnesses the potential of neglected species and promotes agricultural diversification.^4^

While strategies for enhancing food security have often suggested intensification of major cereals, there is an untapped potential to incorporate wild edible plants and underutilized food sources for contribution to food and nutrition security. Reports of wild food species from 91 countries revealed that the wild food species included 1955 plants, 117 fungi, 187 mammals, 156 birds, 21 insects, 30 crustacea, 38 molluscs, 262 fish, 45 reptiles and amphibians, and five other species.^5^ In India, 1403 species of wild edible plants are consumed, with leafy shoots and fruits being the two most eaten wild plant species.^6^ Although access to wild sources of food are declining as natural habitats are destroyed by industrialization, wild plants can indeed provide a significant proportion to the ‘global food basket’, particularly when agricultural productivity is low due to climate events.^7^ Wild food plants are traditionally used mainly in rural populations.^8^ The expansion of the food supply to include neglected and underutilized species is especially important in poor and developing countries. Underutilized and forgotten plants, with good nutritional properties, bioactive potential and health benefits^9^ should be incorporated into the food supply.^10^

The world has a shortage of nutrients. Presently, there has been significant activity in the development of new sources of protein and protein concentrates. Almost 50 years ago there was a request for improving the nutritive value, utilization and availability of food protein, including leaf protein concentrates and single cell proteins.^11^ More recently, unconventional food plants have been explored as protein sources including seeds (*Morinaga oleifera*, *Jatropha curcas*, *Cannabis sativa*), leaves (*Pereskia aceuleta*, *Beta vulgaris*) and stems (*Bambusa vulgaris/Gramineae bamsusoidaea*).^12^ Other unconventional food sources that have been examined for their potential as future protein from the perspectives of nutritional profiles and environmental impacts include terrestrial foods such as cultured meat, mycoprotein (*Fusarium venenatum*), black soldier fly larvae (*Hermetia illucens*), housefly larvae (*Musca domestica*) and mealworm larvae (*Tenebrio molitor*), and aquatic foods including chlorella (*Chlorella vulgaris*), spirulina (*Arthrospira platensis*), sugar kelp (*Saccharina latissima*) and mussels (*Mytilus* spp.).^13^

Microorganisms are unconventional food resources. Edible microbial biomass and products including yeast protein,^14^ fungal protein and mycoprotein,^15^, ^16^ microalgal biomass, edible microorganisms and single cell proteins^17^, ^18^ are becoming attractive alternatives to conventional foods and a sustainable option to address food demand.^18^, ^19^ Some fungi have traditionally been a food source and used in the production of fermented food and beverages.^20^, ^21^ Mushrooms and yeasts have either been eaten directly or used as a food component for thousands of years. More recently, there has been interest in fungi and other microbial proteins as alternative sources of protein and also in mycoprotein as a complete food source.^22^

Physical, chemical and biological unit operations are used in food processing. Resilient food processes which ‘provide transformations to retain or enhance inherent food characteristics, ensuring food safety, nutritional and functional quality, minimizing resource consumption and waste generation, while respecting consumer preferences, acceptance and needs’,^23^ is becoming an increasing important component of sustainable food systems. Gentle (based on the German word ‘schonend’) processing aiming for nutritional and functional preservation of products are being used to describe technologies which are low in resource requirements (e.g., energy, water, minimum waste) in response to fulfilling consumer preferences, acceptance and needs.^24^, ^25^ Radio frequency, microwave, ohmic and infrared heating have been listed as novel thermal processing techniques and cold plasma treatment, ultrasound processing, high pressure processing (HPP), pulsed electric field (PEF), irradiation and pulsed light treatment as non‐thermal techniques. Among those, cold plasma, PEFs, cavitation technologies such as ultrasound, high pressure and ozone processing have been considered as gentle processes.^26^ The principles of action, status, advantages, disadvantages, and opportunities of some non‐thermal processes (fermentation, irradiation, by high pressure, PEFs, cold atmospheric plasma, ultrasound, ultraviolet light) have been previously compared.^27^

The role and scope of adaptive, fitting, robust and small‐scale food technologies (appropriate technologies) was spelled out as ‘the best technology for a given market … after taking into account the constraints’.^27^ Appropriate food technology considers the complete production‐marketing system and optimizes the total system rather than just the technical components.^28^ An example of appropriate processing technology is the village texturizer, promoted by the Meals for Millions Foundation.^29^, ^30^ The village texturizer was an example of a small‐scale, appropriate technological processing unit. The original definition of appropriate food technology,^28^ encompasses the entire food system rather than just parts of it and this approach has been repeated in the quest to move from simplified linear food value chain approaches to a more complex food web system.^31^

**Figure 1:**
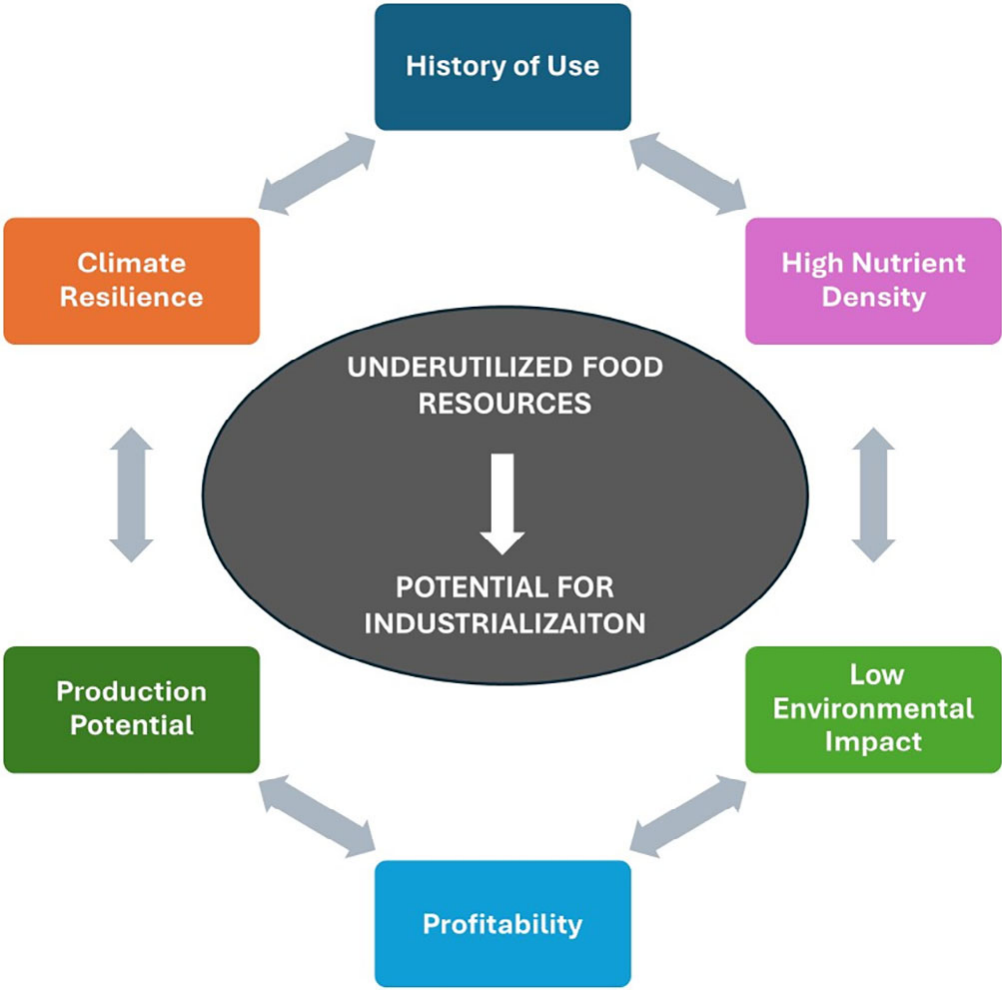
Desirable features of underutilized food resources that help their entry into the food supply.

It is evident that all available food sources need to be utilized and converted into safe, functional, and consumer‐oriented foods. This extends to applying resource‐efficient, gentle, and resilient food technologies. This article discusses the global food demand and the importance of responsible management of the world's ecosystems from the perspective of expanding the diversity of our food supply for improved food security. It examines the development of some promising undervalued and underutilized food sources, combined with the use of traditional and resilient processing technologies for improving food diversity for more sustainable diets. The underutilized food sources reviewed include land‐based plants (grasses and leaves, and plant roots), foods from aquatic sources (seagrass, seaweed and microalgae) and edible fungi (yeast and mushroom). These were selected as they met one or more of the following criteria: history of use, high nutrient density and climate resilient, with potential to improve diversity in a culturally acceptable way. From a production viewpoint, some of the factors that need to be considered include growing and harvesting cycles, crop yields, adaptability to climate and soil conditions, and profitability of the crop (input costs and market prices). For these underutilized new food resources to be added to the diversity of the food supply they should have the potential to be exploited commercially on a large scale. Figure 1 shows some of the criteria that are important for facilitating their path to integration with the current food supply.

## PROCESSING OF UNDERUTILIZED FOOD RESOURCES

Food processing is an integral part of the conversion of agricultural produce into safe and consumer acceptable foods. An important consideration of food processing is the choice of the process that enables minimization of loss of nutrients and bioactive components. Both traditional and emerging food processing methods are currently used in industry.

**Figure 2:**
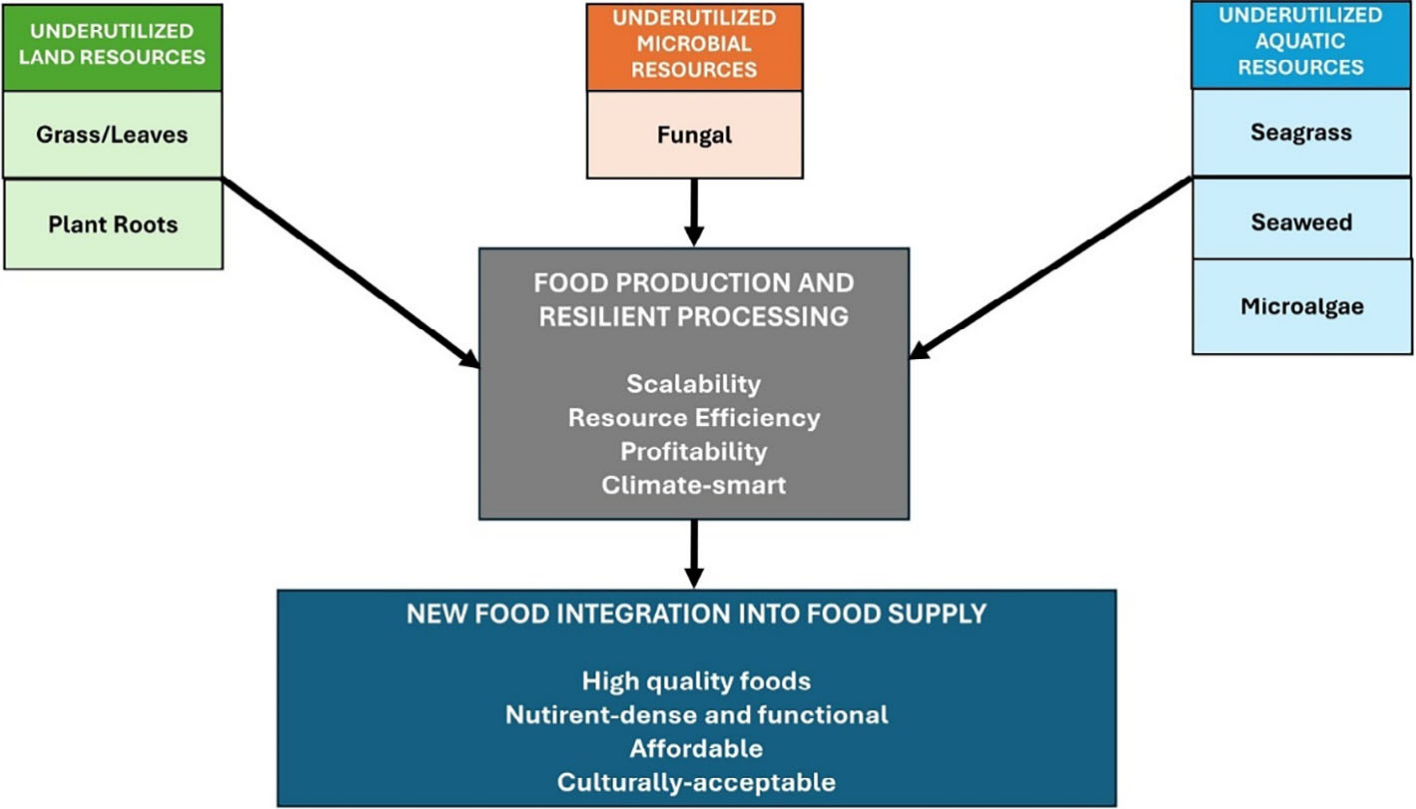
Integral role of food production and processing for increasing availability of underutilized food resources.

A historical perspective on the use of food processing highlighted the use of various mechanical processes (e.g., cutting, pounding, grinding, milling), thermal processes (e.g., drying, cooking, smoking, roasting, baking, cooling and freezing) and biological processes (e.g., fermentation, enzyme processes), chemical processes (e.g., osmotic processes, pH alteration) through the ages for preservation and their conversion into palatable foods.^32^ New non‐thermal processing technologies have been applied to a range of food produce.^33^ Non‐thermal processes and traditional low energy methods (e.g., fermentation) which use less energy than thermal and mechanical processing methods should be considered as processing options for new and underutilized food resources. Figure 2 illustrates the pathway to new food introduction into the current food supply.

Underutilized food sources and waste from processing of underutilized foods may be used for extraction of bioactive compounds. Emerging food processing techniques that may be used to assist extraction include ultrasound, microwave, enzyme, supercritical fluid and pressurized liquid extraction and combinations.^34^, ^35^ Some recent developments on underutilized foods discussed in this review are given in Table 1.

**Table 1:** Recent developments in processing of selected underutilized food resources

Processing of underutilized food resources
*Grasses and leaves* • Pretreatment with pulsed electric fields, ultrafiltration to increase juice/protein yields and reduce energy input needs; optimization of plant source combinations for nutrient improvement^36^, ^37^
*Plant root crops* • Reduction of antinutrients/increase of bioavailability of antioxidants by appropriate processing (e.g., thermal treatment, soaking, fermentation; pulsed electric fields, ultrasound, microwave use possible emerging technologies)^38^
*Microalgae* • Use of emerging technologies for extraction of bioactives (e.g., ultrasound extraction pressurized liquid extraction, pulsed electric fields, supercritical fluid extraction, microwave), purification, and potential human health effects.^39^ Use of protein in food formulations and supplements^40^ *Seaweeds* • Increased awareness of culinary potential of ‘sea rice’/ ‘sea wheat’, improved sensory properties, dried and ground seaweeds and extracted hydrocolloids as functional foods and food ingredients^41^, ^42^
*Yeast* *•* Advanced fermentation processes (waste as substrates, upscaling) and modified yeasts (for bioactive agent generation)^14^, ^43^
*Mushrooms* • Submerged culture fermentation processes, photovoltaic driven continuous glasshouse production/processing, modern preservation techniques (cooling, packaging/coating, surface treatment with ozone, ultraviolet light, pulsed lights, ultrasound, cold plasma, pulsed electric fields, high hydrostatic pressure)^44^, ^45^

## UNDERUTILIZED PLANT SOURCES

The plant sources discussed include grasses and leaves, and plant root crops. Selected examples of the most promising candidates for upscaling are discussed, along with processing options for conversion into foods and technologies for protein extraction. It is recognized that other plant‐based sources (e.g., wild fruits, plant shoots) also have potential but are not covered here. A review of many other neglected and underutilized plant sources that help promote sustainable agricultural practices has been published.^46^

### Grasses and leaves

Early attempts to promote grass as human food were dampened by arguments about how much grass can be safely eaten over prolonged periods because of its high fiber (cellulose) content.^47^ Grasses and leaves are now being viewed as a potential source of protein to fill the shortfall in protein demand from conventional sources. The recognition of leaves as a protein source by Ereky in 1917 led to the establishment of the ‘Green Fodder Mill’, the first green protein biorefinery factory, in Hungary and England for the production of animal feed.^37^ One of the pioneers (NW Pirie) of leaf protein research during the Second World War reported on the history and the scale up processes for leaf protein recovery including sugar beet tops or waste from pea canning.^48^ About 50 years ago NW Pirie also suggested that ‘protein content of the discarded haulms of crop plants may be as great as that of the harvested crop’ should be exploited as a source of leaf protein,^49^ although leaf protein was not considered attractive at the time. The leaf protein content for 90 plant species was reported to be 0.72 to 7.02 (% fresh weight) while moisture content of the leaves was 47.4 to 91.9 moisture (% fresh weight).^50^

Alfalfa has been traditionally used as a source of protein for livestock. Alfalfa (*Medicago sativa* L.) was used as plant source for the production of an edible white fraction of leaf protein concentrates about 50 years ago.^51^ However, the use of alfalfa in human food is still limited due to the presence of anti‐nutritional components and sensory qualities.^52^ These authors further suggest that some of the hurdles to the application of alfalfa in human food may be overcome by the use of appropriate processing methods (blanching, isolation and purification of protein) to produce high‐quality protein functional ingredients for food applications as it has a balanced amino‐acid composition.^52^

Stinging nettle, a wild herbaceous perennial flowering plant with spiny leaves, is commonly found in Asia, Africa and Europe. It is edible with many nutritional and medicinal properties^53^ but has been a neglected food source.^54^ At present, it is considered an unconventional food resource. It has been used as a wild vegetable in countries such as Nepal, Georgia, and Romania. Himalayan stinging nettle is a rich source of proteins and minerals.^55^ Nettle leaves contain 0.4–4% protein, 4–7% carbohydrates, 6% dietary fiber, 0.6–9% fat (% fresh weight) and has been used as a green leafy vegetable in many countries where it is used as a spinach substitute.^54^ The protein content of the common nettle (*Urtica dioica*) meal (made by pulping nettle leaves and drying) was reported to be 29.7%.^56^ Nettle leaf protein concentrate has a protein content of 53.4 ± 1.4 g/100 g and may have possibilities for use in animal feed.^57^ Besides its nutritional value, it is a source of bioactive components.^58^ Among the ways the stinging nettle has been processed include blanching to destroy the tiny hairs on the nettle and preserve its color, freezing and drying. Nettle leaf powders that were blanched and freeze dried have been found to have higher retention of bioactives than conventional drying.^59^ Bioactive components extracted from nettle have been used for fortification of functional foods. Examples include the use of extracted carotenoids as an ingredient in egg pasta.^60^ In order to shift the consumption of stinging nettle into the wider food supply, more controlled cultivation techniques are necessary. Cultivation in open fields is an option but the increasing number of extreme weather events makes management of the crop challenging. An alternative that is being considered is the production of stinging nettle in glasshouses with the use of modern hydroponics.^54^

Now leaf plant leaf proteins (including Rubisco) are becoming more recognized as a sustainable and alternative source of protein, with unique functional proteins for use in food product formulation.^61^ Rubisco, a major component of soluble leaf protein has many desirable functional features, including reduced allergenicity, enhanced gelation, foaming, emulsification and textural properties.^62^ Data on comparison of nutritional values of leaf protein concentrates with 12 common food proteins revealed that leaf proteins compared favorably with high quality proteins. The estimated biological values of leaf proteins were generally lower than those of egg and egg white but higher than beef, casein, soybean, yeast, gluten, zein or wheat flour.^63^

Processing grasses and leaves into protein concentrates has involved the use of conventional techniques for isolation of protein. Protein precipitation and separation have included the use of alkaline extraction, isoelectric precipitation (pH 3.0–5.0), ultra‐filtration, thermal coagulation or microwave‐assisted thermal coagulation and flocculants.^37^, ^61^, ^62^ Juice extraction from leaves and grasses, including waste products such as potato haulms or sugar beet tops has been carried out with screw expellers, screw presses, crushers, mills, belt presses, V‐press, and sugar cane rollers. In addition, the use of PEFs, microwave, ultrasound or enzymes have been applied to aid plant juice liberation.^36^, ^37^, ^61^ Recently, perennial rye grass and white clover were examined as plant sources. Processing involved combining a large screw pressing facility with an industrial PEF system (1.1 kV/cm, 305 Hz, 5 μs, total specific energy requirement of 1.53 kJ/kg with an estimated mass flow of 200 kg/h). The PEF treatment increased the yield of pressed juice and crude protein by about 25% and 31% respectively.^36^ Biorefining, using mechanical and natural fermentation techniques, has been combined with organic production of crops (alfalfa, clover, and grass–clover mixture) to increase the commercial value of these crops.^64^ Juice obtained from green biomass (grass juice) has been used as a substrate for the fermentation of microbes for the production of microbial protein.^65^

The examples given highlight the nutritive value of new foods from leaves, the use of leaves as a source of bioactives, and as a novel food ingredient in food. Recent research has confirmed that other sources of leaf warrant further exploration, with growing market opportunities. One of these is the leaves of *moringa olifera* as a new raw food material but further work is needed to overcome the bitter and astringent taste of the plant, to determine toxicological properties to establish safety of ingestion, the functional properties of foods containing these leaves when used in formulations,^66^ and the bioaccessibility/bioavailability of the nutrients/bioactives in leaves.^67^ The other is coffee leaves as a source of bioactives and a food ingredient. More research is needed to bring more coffee leaf ingredients to market despite the emerging patents relating to its application as therapeutic agents and the existence of coffee leaf beverages in some countries^68^ and new insights into the use of coffee leaf powder as a partial replacer for wheat flour in rusks.^69^

### Plant root crops

Plant root foods and root crops have been used by ancient civilizations. Plant roots and rhizomes were a major sources of carbohydrates and energy for hunter‐gatherers in Europe.^70^ Starchy roots and tuber crops (e.g., cassava, sweet potato, yam, cassava, taro, potato) are important part of the human diet^71^ and for food security in many countries.^72^ From the total 1403 edible wild plant species (184 families) identified in India, 219 (70 families) belong to underground parts,^6^ demonstrating the large still mostly untapped potential food sources. There are numerous edible wild carrot varieties still underutilized. For example, it has been reported that the Macaronesian islands (Azores, Madeira, Selvagens, Canary, and Cabo Verde archipelagos) constitute an enormous reservoir of wild carrots and demonstrated the diversity patterns of wild carrots in the western Mediterranean region.^73^ Processing of root crops which has been applied to the conventional crops may be used to expand the diversity of root crop products in the food supply. Starchy roots have been subjected to fermentation for conversion in various beverages and food products.

Carrot is a popular root throughout the world due to its high concentration of carotenoids, anthocyanins, dietary fiber and other functional properties.^74^ Carrots are a good example of a root crop that has been commercially processed into a variety of products (e.g., canned or dried products, juices and concentrates, beverages, candy preserves and intermediate moisture foods).^74^ Carrots were among the earliest food products subjected to PEF treatment.^75^ Carrots have been used for production of shelf‐stable nutrient dense powders and extruded snacks.^76^ The processing of carrots into powders increases carotenoid bioavailability.^77^ Fermentation of carrot juice by lactic acid bacteria increases the carotenoid and fiber contents.^78^ An increase of total carotenoid content of carrots of up to 11.3% after PEF treatment (1.85 kV) has been reported.^79^ HPP of fresh carrots resulted in hardness losses of 5% to 50% at 100 to 300 MPa (initial temperature of 20 °C). Textural changes were associated with turgidity loss due to cell wall breakage.^80^ It would be of interest to explore the benefits of high tissue flexibility after PEF or HPP treatment (e.g., reduced cutting losses, reduced tissue breakage).

Cassava is an adaptable crop with tolerance to low soil fertility and high recovery from pests and diseases. It is widely cultivated in the lowland tropics of Africa (Nigeria) and South America (Brazil), and also found in many parts of Asia. It is less affected by climate conditions than other major food crops,^81^ making it an option in the face of climate change. Cassava is a starch rich root crop. Cassava has been processed into flour, and unfermented and fermented food products for hundreds of years. However, there are toxicity and safety issues with cassava, although processes (washing, drying, fermentation, grating, boiling and chemical treatments) have been used for detoxifying cassava.^82^ Fermented cassava food and beverage products include *gari*, *lafun*, *fufu*, *wanghe*, *attieke*, *tape*, ‘*cheese*’ *bread*, *beju*, *peujenum*, ‘*coated peanut*’, and cassava beer.^83^, ^84^ Traditional processes have now been applied in industrial settings,^84^ overcoming some of the issues with product safety and quality in traditional village practices. Commercially, cassava roots have been used in baked goods where cassava flour may be used to substitute wheat flour. Cassava has been also processed for starch production.^85^

Yam is a crop that is a major food and income source for many communities in tropical and subtropical regions, especially so in West and Central Africa.^86^ There are challenges in intensification of yam cultivation. Integrated soil fertility management has been used to improve yam cultivation.^87^ There is also fermented yam flour (*amala*) and a fermented food product (*poi*).^83^

## UNDERUTILIZED MARINE SOURCES

In this section three underutilized marine sources (seagrass, seaweed and microalgae) which have history of use are discussed. These have a history of use as food, with potential to contribute more to future food security and sustainability. Seagrass has been used as a food source,^88^, ^89^ although processing information appears to be limited. There is significant growing interest in seaweed^90^ and microalgae^91^ sustainable future food sources.

### Seagrass

Seagrass is a marine plant that produces flowers, fruit and seeds. There are extensive meadows of seagrass in intertidal and shallow water environments in all continents except Antarctica.^92^, ^93^ Most of the literature on seagrass relate to its ecology and role in maintaining aquatic environments and protecting biodiversity.^93^, ^94^

Seagrass is a rich source of nutrients. Eelgrass (*Enhalus acoroides*) has 13.4% protein, 2% nitrogen‐free extract, 1.4% lipid, 20.3% crude fiber and 26.63% ash.^89^ Manatee grass (*Syringodium filiforme*) (Kützing), from a lagoon in south‐eastern Mexico, was found to have 10.43% protein, 45.37% nitrogen‐free extract, 2.43% lipid, 19.43% fiber and 23.43% ash.^88^ Historically, they have been in the diets of some coastal populations. For example, the seeds of eelgrass (*Zostera marina* L.) are an important traditional food of the Seri Indians of Sonora and New Mexico. The harvested eelgrass is dried and the grain is separated, ground and made into flour, which is made into gruel.^95^ The fruits of seagrass has been eaten by the traditional people living on the cost of Australia and Philippines, and there is anecdotal evidence of seagrass being eaten by fisherman.^96^ The role of seagrass as a new potential source for human nutrition and farming of seagrass may provide a sustainable food source for future food security. The microbiological quality of the seagrass and the presence of heavy metals need to be examined and they have to meet safety regulations for food.

### Seaweed

Macroalgae (seaweed) are multicellular large size algae. They are classified into three major groups: red algae (*Rhodophyta*), brown algae (*Phaeophyta*) and green algae (*Chlorophyta*), with estimates of 6200 different red algae, 1800 brown algae, and 1800 green algae.^97^ Seaweed has been used for several centuries as food in countries such as China, Japan, Korea, and Mexico and is now finding its way as an exotic component of foods in Europe. Macroalgae are low in calories, rich in soluble dietary fibers, proteins, minerals, vitamins, phytochemicals and polyunsaturated fatty acids.^98^ Green and red seaweed contain more protein (10–47% dry weight) than brown seaweed (5–24% dry weight). The lipid content (0.79–7.87% dry weight) comprises a significant portion of ω‐3 and ω‐6 polyunsaturated fatty acids. The dietary fiber (36–60% dry weight) has a high soluble fiber content (55–70%), the mineral (8–40% dry weight) comprises essential minerals (sodium, calcium, magnesium, potassium, chloride, sulfate, phosphorus) and micronutrients (iodine, iron, zinc, copper, selenium, molybdenum, fluoride, manganese, boron, nickel, cobalt). In addition, algae are an excellent source of vitamins (A, B_1_, B_12_, C, D, and E, riboflavin, niacin, pantothenic acid and folic acid). The health properties of seaweeds are also due to the content of bioactive molecules (polyphenols, polysaccharides and sterols).^99^ Seaweed is now grown for human consumption, as new markets for the product are emerging because vegans and vegetarians are introducing seaweed into their diets.^99^

Fresh seaweed (*Laminaria* and *Undaria*) and dried seaweed (*Porphyra*, *Laminaria*) have been cooked and processed for foods in many ways (e.g., frying, stewing, steaming, boiling).^100^ The processing of edible seaweed and seaweed extracts (e.g., hydrocolloids, ant‐oxidants, omega‐3 oils) enables it to be incorporated into a range of foods where they contribute to nutritional, sensory and health promoting properties of food.^101^ Processing seaweed can improve the safety, utilization and storage stability of seaweed products. From a microbial perspective, pathogenic *Bacillus* spp., *Vibrio* spp., and *Aeromonas* spp. as the main inherent bacteria, are of concern for the food safety of seaweed. Microbial growth can be controlled by drying, thermal processing, fermentation, freezing, salting, gamma irradiation, and emerging technologies such as HPP.^102^ Various microbial cultures (lactic acid bacteria, yeasts and filamentous fungi) have been used for fermentation of seaweed to enhance sensory and health promoting properties. Some of the fermented seaweed products (fermented seaweed broth, sauce and extracts) have enhanced levels of antioxidants, γ‐aminobutyric acid and bioactive peptides.^103^ Many extraction techniques have been used to isolate bioactive components that are present in seaweed. A comparison of the advantages of newer extraction techniques have been compared to traditional seaweed processing methods for extraction of oils and volatiles.^104^

Consumers in Asia (Japan, China, Korea, Indonesia) and Pacific Islands have traditionally eaten seaweed fresh or in dried forms.^105^ The functional and technological properties of seaweed make them attractive to be incorporated as an ingredient in formulated foods, including meat products,^106^ dairy products such as yoghurt and quark,^107^ pasta,^108^ bread,^109^ bakery products, cereal bars and seafood products. They are incorporated into meat products to enhance their nutritional and textural properties.^99^, ^110^, ^111^ A study of Australian consumers indicated that people with higher education, health‐conscious snackers and adventurous in their food choice were early adopters of seaweed‐based products.^105^ While the increased use of seaweed and seaweed ingredients in food is promising, some challenges remain in wider acceptance of seaweed in food products, especially in the European markets due to their sensory impact on the food, the lack of consumer understanding of the health benefits of seaweed and market barriers related to food safety and quality.^112^ Despite positive attitudes of consumers to seaweed consumption around health and sustainability, another challenge is the lack of consistency in their composition and quality which affects sensory attributes.^113^ Currently seaweed is not included in dietary recommendations or national food strategies in Western countries. Combining traditional knowledge about food preparation practices for seaweed with a science evidence‐based gastronomical development will help make seaweed more visible in the planetary menu.^114^

### Microalgae

Microalgae are a diverse group of unicellular microscopic organisms. There is an extensive biodiversity of microalgae. There are widely varying estimates of the number of algal species, ranging from 300 000 to more than one million.^115^ Microalgae has been used as foods by some populations for millennia (e.g., edible blue‐green algae *Nostoc* in China and *Arthrospira* (*Spirulina*) in Chad and Mexico), and also as a delicacy from South America to Asia.^116^

The chemical composition of microalgae varies greatly across different microalgae classes, species, and strains, with even the same strains having different compositions depending on culture conditions. The reported composition of a selection of different microalgae (% dry weight) showed they contain 9.7–71% protein, 4–64% carbohydrates, and 1.1–38% lipid. They are also a valuable source of vitamins and pigments.^117^ More recent reports have stated that the crude protein content of microalgal biomass ranges from 30% to 80%.^118^ Cultivation of algae for food and feed is relatively recent (in the last few decades). Today the microalgal species which account for most of the global microalgal production include *Spirulina*, *Chlamydomonas reinhardtii*, *Chlorella*, *Haematococcus pluvialis*, *Dunaliella salina*, *Schizochytrium* and *Nannochloropsis*.^119^

Various methods have been used for processing microalgae. Microalgae biomass is usually dried after harvesting. Drying methods examined include infrared drying, drum drying, air drying, spray drying, freeze drying and sun drying. Conventional heat treatment processes (boiling, autoclaving) have been applied to ensure safety of microalgae for human consumption.^117^, ^120^ Ultrasound has been shown to enhance the protein digestibility and biological value of microalgae (*Chlorella vulgaris*).^120^ The high protein content of microalgae makes it an attractive alternative protein source. A range of methods for protein extraction has been applied including conventional aqueous extraction methods followed by ultrafiltration after pretreatment to disrupt the cell walls (e.g., using bead milling, ultrasonication, microfluidization, enzymic disruption, PEF and microwave‐assisted extraction).^121^ Fermentation of microalgae biomass was reported as a means for generation of functional foods or ingredients.^122^

Microalgae products are unconventional foods. There should be testing of the microalgae and data on quality to ensure they are safe and free from hazardous physical and chemical substances (e.g., heavy metal contamination) and pathogenic microorganisms that are harmful to human health.^117^ There is slow market penetration of microalgal products. Microalgae may be added as a whole dried biomass/microalgal extracts^91^ into food. Microalgae has potential applications as an ingredient in a range of food products (bread, baked goods, burgers, extruded snacks, dairy products).^91^ The social acceptance of microalgae as a food is still a hurdle for market entry. Increasing consumer understanding and the need for improvement of processing technology for microalgae such as decolorization, flavor improvement and extrusion cooking and three‐dimensional (3D) printing may be applied to improve the quality of microalgal products.^119^

## UNDERUTILIZED FUNGAL FOOD SOURCES

The consumption of edible fungi have been part of the food supply since ancient times and played a major role in the health of communities. There has been increased interest in the use of edible fungi as a source of protein^123^, ^124^ due to the world shortage of protein, greater demand for production of sustainable sources of protein and rising interest in alternative meat‐analog products. In this section, two edible fungal food sources (yeast and mushroom) are discussed. At present, they are still underutilized food sources. However, interest in them as more mainstream food sources has been growing due to concerns about food and protein security and the recognition that they are part of the solution for improved sustainability.

### Yeast

Yeast has been used by humans for bread and beverage making for at least 4500 years and was later developed to produce concentrations of yeast during the 10th century. *Candida utilis* soups were provided in Germany during the First World War and the Second World War. Spent brewer's yeasts are a rich source of protein, minerals, vitamins and nutraceuticals, and used in supplements and as fermentation substrate.^22^, ^125^ In the 1960s the production of microbial protein from yeast fed with a by‐product of oil refineries was developed. Protein‐vitamin concentrates were produced by microbes.^126^

*Saccharomyces cerevisiae* has a ‘Generally Regarded as Safe’ (GRAS) status for food products. *Yarrowia lipolytica* has been approved via the Novel Food Regulation (EU 2017/2470) where its use is restricted to food supplements.^22^ The yeast species *Kluyveromyces marxianus* has been examined for production of enzymes, single cell protein, aroma compounds and ethanol, and categorized as GRAS.^127^ *Candida utilis* species has GRAS status. It fulfills requirements of the fodder yeast^128^ and has been used in animal feed and as a seasoning agent in vegetarian food.^14^

The yeast biomass is an excellent source of proteins, the B vitamins, contains high levels of carbohydrates (31–51%, dry weight), is low in lipids (4–7%, dry weight) and trace minerals.^14^ *Yarrowia lipolytica*, an oleaginous yeast, cultured on various fatty substrates is a good source of protein (30–50% dry weight) and can accumulate intracellular lipids (> 40% cell, dry weight) and assimilate vitamin B_12_.^14^ The incorporation of yeast in food products is limited due to its high content of nucleic acid, its low digestibility and bitter taste if the yeasts are not processed. Processing, with the use of enzymes, have been used to reduce the nucleic acid content of yeast.^129^ Consumer acceptance and resistance has still been considered as limiting factor for the wide spread use of yeast protein in human diets.^14^ This is despite the production process being highly scalable, efficient, sustainable and economic.^130^ The formulation of foods with yeasts have to be further developed, to facilitate the adoption of yeast as an ingredient to suit the consumer preferences in different countries. This was exemplified in a recent study on the use of proteins from torula yeast as a vegan spread alternative.^130^

### Mushroom

Mushrooms are the fleshy fruiting and spore bearing body of a fungus.^44^ There is considerable potential for edible mushrooms to be exploited as a wholesome food. While the composition of the major industrialized mushroom is known, there are many wild and under‐exploited ones for which nutritional data are limited.^131^ An African study^132^ identified 480 species of wild edible mushrooms and found them to provide nutritious food for humans.

Mushrooms, particularly the wild ones are known as source of vitamins, especially vitamin D (the only non‐animal source), have high mineral content, average protein contents of 20% to 30% (dry weight basis), high essential amino acids levels, low carbohydrates, fiber and fat and additionally have chemicals with antioxidant properties.^132^, ^133^ They possess considerable nutraceutical and therapeutic properties including antitumor and immunomodulatory actions as well as antiviral, antibacterial and anti‐inflammatory properties. Exposure of mushrooms to sunlight or ultraviolet radiation stimulates the conversion of its ergosterol into vitamin D_2_.^134^, ^135^ This offers a potential of targeted processing to increase nutritional values (e.g., sun drying, ultraviolet surface decontamination).

Due to high moisture contents (85–95%), high respiration rates (200–650 mg/kg/h) and high initial microbial counts (5–12 CFU/g), fresh mushrooms are highly perishable.^44^, ^133^ Cooling (3–5 °C), packaging, coating, chemical treatments (washing with electrolyzed water, citric acid solutions) are the most common postharvest treatments. As for processing methods, these include drying, gamma irradiation, ultraviolet treatment, cold atmospheric plasma, high hydrostatic pressure and canning,^44^, ^133^ use of modified atmosphere packaging and active packaging to postpone senescence, and thermal regulating material and phase change materials in combination with intelligent evaluation technologies.^136^ Most studies concentrate on preservation effects and quality parameter (color, texture, flavor) retention, while limited information exists on the impact of postharvest and processing techniques on nutritional quality maintenance or improvement.^44^ Considering the high diversity of still untapped biological and nutritional resources mushrooms can provide,^132^ exploring the potential effects of resilient processing techniques,^23^ their integration with food production and with consumer preferences, acceptance and needs, seem highly relevant. However, there are still challenges for consumer acceptance of mycoprotein due to lack of sensory appeal and understanding of health effects.^137^

## CHALLENGES AND PERSPECTIVES

The introduction of new and underutilized foods into the market as whole foods or as ingredients in formulated food is a challenging and complex process. Food hazards and food safety gaps must be addressed by food safety agencies and food manufacturers.^138^ New foods that are nutrient‐dense, climate resilient, profitable and have good environmental sustainability credentials are more likely to be accepted. However, unless there is a pathway to consumer acceptance, the new food will not be consumed. The main factors that need to be addressed when introducing new foods into the food supply from a consumer viewpoint are overcoming food neophobia, unfamiliarity of the product and sensory properties. On the positive side, there are also drivers of acceptance if products are affordable, and perceived as healthy and environmentally sustainable.^139^ New and underutilized foods discussed have the same similar general challenges relating to consistency of the raw material, choosing species/variety of new foods for efficient, sustainable, productive and profitable production systems, selection of the appropriate postharvest processes for preservation and conversion into safe, nutritious and palatable food products, complying with food regulations for novel foods, overcoming consumer acceptance issues and market entry and gaining sustained market share. Life cycle assessments using standardized procedures will need to be carried out and validated. Each new and underutilized food will face different challenges. Some of these are summarized in Table 2. However, ultimately it is the economics and sustainability of the whole value chain that will determine the success of bringing new foods into the market.

**Table 2:** Challenges and perspectives for underutilized food resources

Challenges and perspectives
*Grasses and leaves* ^37^, ^140^
• *Challenges*: Limitations due to land use conversions and degradation, anti‐nutritive factors (e.g., oxalic acid, tannins, phenolic compounds) removal, sensory property improvements, safety assessments and approval, consumer acceptance
• *Perspectives*: Grasslands create and stabilize fertile soil, store oxygen and increase biodiversity and food supply, plant‐based food waste use and reduction, cost effective extraction and functionalization with emerging technologies (e.g., pulsed electric fields, ultrafiltration, flocculation, super critical carbon dioxide, microwave, fermentation), acceptance as plant‐based meat and egg analogs
*Plant root crops* ^10^, ^38^
• *Challenges*: Antinutrients removal (e.g., oxalates, phytates, tannins), soil contaminants uptake (e.g., metals, pesticides, microplastics, pharmaceutical residues, nitrates), need for standard assessment methods (e.g., microplastics) and permissible standards for uptake
• *Perspectives*: Low‐cost dietary energy sources (e.g., taro roots) and staple foods with high nutritional potential, regional food security improvements, unique starch and fiber sources due to emerging technology processes (e.g., pulsed electric fields, ultrasound) and scale up development
*Seagrass* ^141^, ^142^ • *Challenges*: Rapid decline of seagrass meadows around the world, seagrass not currently widely recognized as conservation priority, • *Perspectives*: Management of seagrass ecosystems to sustain food security for fishing communities and poor populations *Microalgae* ^91^, ^143^ • *Challenges*: High production costs, lack of data on real economic and environmental benefits, safety concerns, low consumer acceptance • *Perspectives*: Promising source of macronutrients (protein, polyunsaturated fatty acids, polysaccharides), bioactives, and pigments *Seaweed* ^144^, ^145^
• *Challenges*: Reduction/losses due to coastal developments, aquaculture, low water quality, need for means to encourage consumption and related risk assessments
• *Perspectives*: Nature‐based solution for greenhouse gas mitigation and water filtration (seagrass), protection of coastal areas, alternative food, food ingredients and fiber sources, potential for extraction of bioactive compounds, potential for innovative seaweed‐based food sources, increased subsistence and sustained food security for coastal regions, reduction of land pressure for food generation
*Yeast* ^14^, ^43^
• *Challenges*: Increasing awareness of environmental benefits, attention to allergic substances and microbial contaminants, reduction/removal of antinutritional nucleic acid
• *Perspectives*: Upgrading food waste and processing side streams, added value application, replacement of less sustainable conventional protein and nutrient sources, decrease of environmental pressure on land and water use, improved product safety via innovative processing (e.g., enzyme or pulsed electric field assisted reduction/removal of nucleic acid); availability of non‐seasonal food sources
*Mushroom* ^45^, ^146^, ^147^
• *Challeng*es: Continuous large‐scale production, shelf‐life extension needs, avoidance of accumulation of heavy metals and radioactive isotopes, lack of standard protocols to guarantee quality, safety, efficacy and shelf‐life extension • *Perspectives*: High bioactive food source, renewable energy driven continuous production and preservation (e.g., glasshouses with controlled substrates, advanced packaging), detoxification of contaminated sources (e.g., fermentation), new food sources due to mushroom diversity exploration, redesign food supply chains for environmental sustainability

## RECOMMENDATIONS FOR EXPANDING FOOD SOURCES

Goal 2 of the United Nations (UN) Sustainable Development Goal is to ‘End Hunger, achieve food security and improved nutrition and promote sustainable agriculture’.^148^ Rediscovering and re‐introducing neglected and underutilized species will help alleviate hunger.^149^ Neglected and underutilized crop species should be recognized as having an important role in improving the diversity of diets and fighting hunger and malnutrition.^139^ Key to the utilization of the neglected food sources more sustainably is to use of a comprehensive approach to expanding the biodiversity of our food supply and application of resilient processing technologies. Selected strategies and actions that will help expand the food supply and improve food security and sustainability are given in Table 3. It is important to seize on the opportunity for increasing diet diversity to expand our food supply and minimize the use of the world's diminishing resources for ensuring food security and sustainability in future.

**Table 3:** Selected recommendations for expanding our food supply for improved food security and sustainability

Strategies to facilitate expansion of our food supply
*Increase consumer awareness*
• Raise consumer awareness for improved food diversity
• Increase food processing and preparation literacy towards neglected and underutilized food sources (NUFs)
• Increase consumer awareness about food products from more sources
*Improved basis for informing research strategies and policy*
• Shift consumer demands towards more sustainable and diverse diets
• Increase funding and expand research on resilient processing of NUFs
• Improve interlinking between food production, processing, distribution and consumption of NUFs
• Promote energy and resource efficient technologies
*Opportunities for action*
• Redesign food supply chains for environmental sustainability
• Increase circularity of food systems and reduce food waste
• Assure minimum loss and waste of NUFs during production, processing and consumption
• Design resource efficient NUFs production systems; Increase resource efficiency of NUFs • Apply resource efficient processes tailored towards optimum nutrient retention and increase • Select best fitting resilient food processes for specific NUFs
• Abolish unsustainable production, processing, transportation, and preparation practices
• Re‐evaluate existing food transportation practices regarding energy and resource efficiency

**Figure 3:**
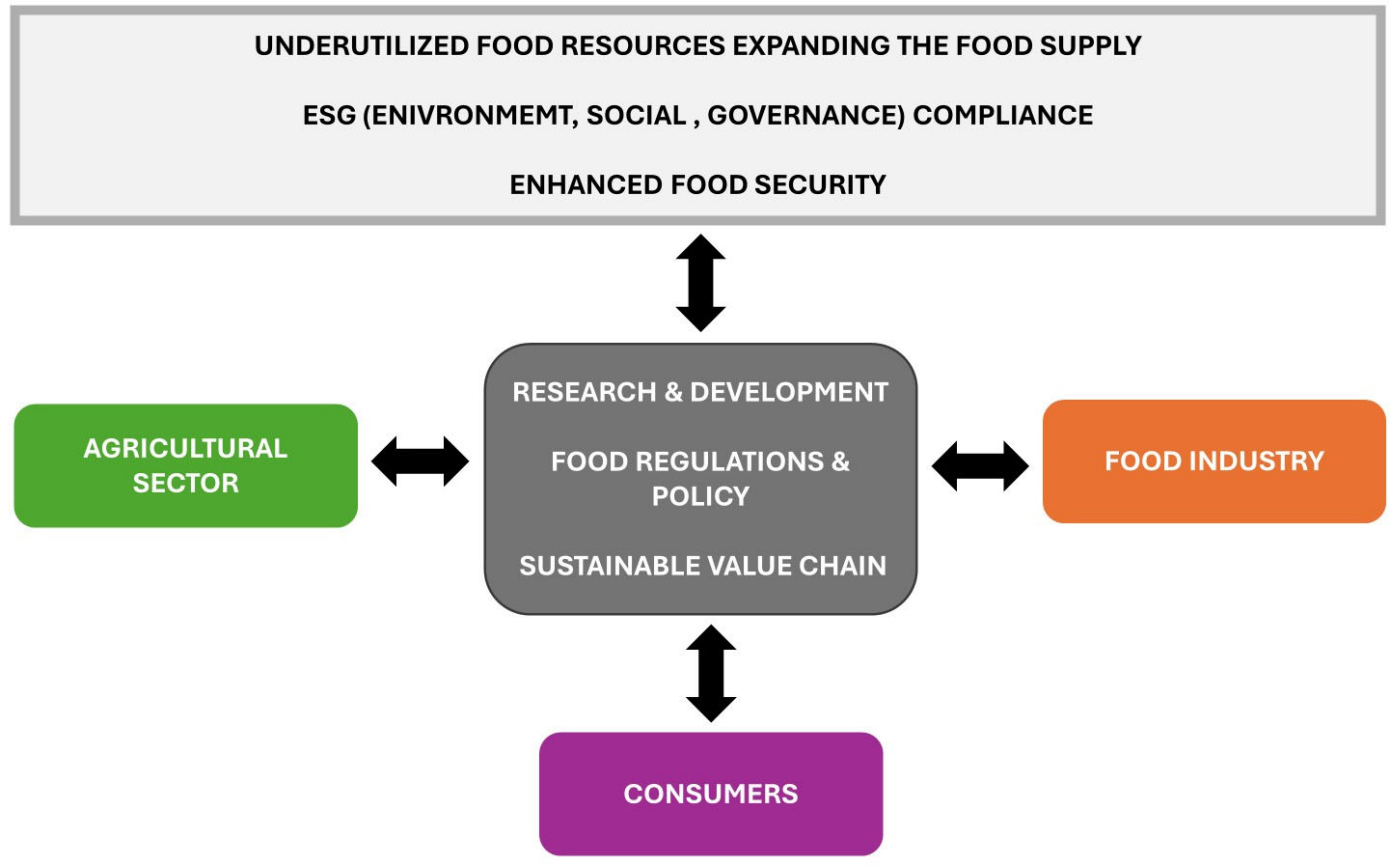
Multistakeholder engagement for expanding the food supply to include underutilized food resources.

The FAO's Strategic Framework calls for ‘better production, better nutrition, a better environment and a better life’ to transform agri‐food systems to be more efficient, inclusive, resilient and sustainable.^150^ Multistakeholder inputs across the food chain are required to provide inputs for successful sustainable food chains. This includes inputs from those involved in production on farm, postharvest handling and storage, food processing and packaging, food regulations, marketing, and consumers (Fig. 3). Decision makers will need to take action to consider the trade‐offs between the need to balance human health, economics and environmental sustainability when introducing new foods into the market. Increasingly the success or otherwise of the adoption of new foods will be led by the multiple stakeholders and adherence to ESG (environmental, social and governance) criteria.^151^ Environmental (greenhouse gas emissions, biodiversity, water and waste management, use of natural resources), social (diversity and inclusion, consumer protection, community support) and governance (business ethics, corruption, accounting transparency, management structure, employee relations) credentials will be required for effective production and manufacture of foods. Momentum is growing for the need for ESG compliance in the food sector and an evidence‐based ESG‐nutrition metrics has been proposed.^152^

## CONCLUSIONS

Neglected and underutilized plant species are considered to have relatively low value for global production and the market. This is because they do not fit the modern standards of uniformity compared to the major cultivated varieties of plant crops.^153^ However, many underutilized plants are often resilient and are important sources of nutrients, with potential to be developed and included in daily diets.^154^ The utilization of the neglected food sources more sustainably requires an approach that expands the biodiversity of our food supply. It is also important to promote the role of food processing in food security, which involves applying appropriate resilient processing technologies for the manufacture of safe, nutritious and culturally acceptable foods.^155^

## AUTHOR CONTRIBUTIONS

Dietrich Knorr – conceptualization. Dietrich Knorr and Mary Ann Augustin – writing, editing, reviewing. All authors have read and approved the final manuscript.

## CONFLICT OF INTEREST

The authors declare no competing interests.
